# MicroRNA-23a-5p Is Involved in the Regulation of Lipopolysaccharide-Induced Acute Lung Injury by Targeting HSP20/ASK1

**DOI:** 10.1155/2021/9942557

**Published:** 2021-08-11

**Authors:** Yi-Fei Chen, Fen Hu, Xian-Guo Wang, Zheng Tang, He-Xiao Tang, Ming Xu

**Affiliations:** ^1^Department of Respiratory and Critical Care Medicine, Zhongnan Hospital of Wuhan University, Wuhan, 430071 Hubei, China; ^2^Department of Respiratory Medicine, The First People's Hospital of Jiangxia District, Wuhan, 430200 Hubei, China; ^3^Department of Thoracic Surgery, Zhongnan Hospital of Wuhan University, Wuhan, 430071 Hubei, China

## Abstract

Inflammation and oxidative stress contribute to the progression of acute lung injury (ALI). MicroRNA-23a-5p (miR-23a-5p) has been reported to regulate inflammation and oxidative stress; however, its role in ALI is still poorly elucidated. Mice were intravenously treated with the miR-23a-5p antagomir, agomir, or the negative controls for 3 consecutive days and then received a single intratracheal injection of lipopolysaccharide (LPS, 5 mg/kg) to induce ALI. Pulmonary function, bronchoalveolar lavage fluids (BALFs), arterial blood gas, and molecular biomarkers associated with inflammation and oxidative stress were analyzed. In addition, murine peritoneal macrophages were isolated and treated with LPS to verify the role of miR-23a-5p in vitro. We detected an elevation of miR-23a-5p expression in the lungs from ALI mice. The miR-23a-5p antagomir was prevented, whereas the miR-23a-5p agomir aggravated inflammation, oxidative stress, lung tissue injury, and pulmonary dysfunction in LPS-treated mice. Besides, the miR-23a-5p antagomir also reduced the productions of proinflammatory cytokines and free radicals in LPS-treated primary macrophages, which were further augmented in cells following the miR-23a-5p agomir treatment. Additional findings demonstrated that the miR-23a-5p agomir exacerbated LPS-induced ALI via activating apoptosis signal-regulating kinase 1 (ASK1), and that pharmacological or genetic inhibition of ASK1 significantly repressed the deleterious effects of the miR-23a-5p agomir. Moreover, we proved that the miR-23a-5p agomir activated ASK1 via directly reducing heat shock protein 20 (HSP20) expression. miR-23a-5p is involved in the regulation of LPS-induced inflammation, oxidative stress, lung tissue injury, and pulmonary dysfunction by targeting HSP20/ASK1, and it is a valuable therapeutic candidate for the treatment of ALI.

## 1. Introduction

Acute lung injury (ALI) is a life-threatening respiratory disorder for which effective therapeutic methods are lacking. Thus, the disease is associated with poor prognosis and high mortality, especially in critically ill patients [1, 2]. During ALI, the pulmonary structure and alveolar-capillary barrier are destroyed, allowing inflammatory cells (e.g., neutrophils and macrophages) to penetrate the lung tissue, where they secrete numerous cytokines that amplify local proinflammatory networks [3, 4]. Overproduction of reactive oxygen species (ROS), which causes severe oxidative damage to intracellular biomacromolecules, including DNA, protein, and lipid, is also implicated in the pathogenesis of ALI. Upon increased ROS, thioredoxin-interacting protein (TXNIP) detaches from thioredoxin and activates the nucleotide-binding domain-like receptor protein 3 (NLRP3) inflammasome, which helps to accelerate the maturation and release of proinflammatory cytokines, such as interleukin-1*β* (IL-1*β*) and IL-18 [5–7]. These findings indicate that inhibiting inflammation and oxidative stress may be potential strategies for the treatment of ALI.

Apoptosis signal-regulating kinase 1 (ASK1) belongs to the ubiquitously expressed mitogen-activated protein kinase family and is involved in the regulation of inflammation and oxidative stress [5, 8]. Recent studies have demonstrated that ASK1 activation contributes to ALI progression, and that inhibitors of ASK1 have potential benefits for the management of ALI [5]. Immanuel et al. found that ASK1 promoted NLRP3 inflammasome priming in macrophages, thereby aggravating the proinflammatory response. Conversely, ASK1-deficient mice had significantly less inflammation and lung injury upon lipopolysaccharide (LPS) inhalation [9]. Besides, ASK1 deletion also decreased hyperoxia-induced inflammation, oxidative stress, and pulmonary dysfunction in mice [10]. These studies identify ASK1 as a critical molecular target in ALI development, indicating that the identification of novel ASK1 inhibitors is greatly needed.

MicroRNAs are short (~22 nucleotides) noncoding RNAs that negatively regulate gene expression at the posttranscriptional level via binding to the 3′ untranslated region (UTR) of target messenger RNAs [11, 12]. Emerging evidence has demonstrated the importance and necessity of microRNAs in pulmonary developmental and pathological processes [11]. Results from Wei et al. indicated that microRNA-377-3p (miR-377-3p) stimulated protective autophagy and suppressed LPS-induced inflammation and lung injury [13]. In addition, Chen and colleagues demonstrated the beneficial role of miR-199a-3p against LPS-induced ALI, showing that miR-199a-3p downregulation aggravated intrapulmonary inflammation and pathological injury [14]. miR-23a-5p exhibits multiple biological functions, such as the modulation of cell proliferation, differentiation, senescence, survival, and oncogenesis [15–17]. miR-23a-5p also regulates inflammation and oxidative stress [18, 19]. Moreover, Liu et al. detected increased miR-23a-5p levels in serum, lung tissues, and macrophages after LPS stimulation and proposed miR-23a-5p as a potential biomarker for sepsis-induced acute respiratory distress syndrome at early stage [20]. However, its therapeutic role in inflammation, oxidative stress, and ALI remains elusive. In the present study, we established an LPS-induced ALI mouse model and investigated the role and underlying mechanism of miR-23a-5p in ALI.

## 2. Materials and Methods

### 2.1. Animals

Male C57BL/6 mice (~25 g) were provided by Beijing HFK Bioscience Co., Ltd. and intratracheally injected with 5 mg/kg LPS (from *E. coli* O111: B4; Sigma-Aldrich, USA) dissolved in 50 *μ*L sterile saline. Mice in the control group received an equal volume of intratracheal sterile saline [7]. To clarify the role of miR-23a-5p, mice were pretreated with miR-23a-5p antagomir (80 mg/kg/day), antagomir negative control (AntagNC), agomir (30 mg/kg/day), or agomir negative control (AgNC) via tail vein injection for 3 consecutive days prior to LPS exposure as previously described [21]. Antagomir, agomir, and negative controls were purchased from RiboBio Co., Ltd. (Guangzhou, China). All mice were euthanized 12 h post-LPS treatment with an overdose of sodium pentobarbital. In the survival study, mice were injected with a lethal dose of LPS (25 mg/kg), and the survival rate was monitored every 12 h [7]. To inhibit endogenous ASK1, mice were treated daily with selonsertib (4 mg/kg; Selleck, USA) for 7 consecutive days prior to LPS injection [22]. In addition, 1 week before LPS treatment, mice were intratracheally treated with recombinant adenoviral vectors (1 × 10^8^ PFU per mouse) carrying short hairpin RNA targeting HSP20 (shHSP20) to knock down pulmonary HSP20 expression or scramble RNA (shScramble) [5, 23]. The mouse HSP20 targeting sequences were obtained from Santa Cruz and then packaged into adenoviral vectors by Vigene Bioscience (Rockville, USA). All animal procedures were approved by the Animal Experimentation Ethics Committee of Zhongnan Hospital of Wuhan University and are in strict accordance with the Guides for the Care and Use of Laboratory Animals published by the US National Institutes of Health (8th Edition, 2011).

### 2.2. Pulmonary Function Measurements

Mice were anesthetized, tracheostomized, and mechanically ventilated using the FlexiVent device (SCIREQ Inc., Canada). Invasive pulmonary functional parameters were collected using the forced oscillation technique and the constant-phase model. Respiratory system resistance (Rrs), elastance (Ers), tissue damping (Gtis), and tissue elastance (Htis) were determined in a blinded manner [24]. Pulmonary function was also noninvasively evaluated using the Buxco system (Buxco Electronics, USA). Respiratory rate, tidal volume, lung compliance, and pulmonary ventilation were detected in anesthetized mice as previously described [7].

### 2.3. Bronchoalveolar Lavage Fluids (BALFs) Collection and Analysis

BALFs were obtained from 3 intratracheal injections of 1.0 mL of cooled phosphate buffer saline (PBS), which were then centrifuged at 200 g for 10 min at 4°C with the supernatants collected for total protein quantification using a BCA protein assay kit (Sigma-Aldrich, USA) [25]. The cell pellet was resuspended in 1 mL PBS. Total leukocytes were counted using a hemocytometer, and differential cell counts were calculated by Wright-Giemsa staining under the standard hematology criteria [6, 13].

### 2.4. Lung Wet-to-Dry (W/D) Weight Ratio

Fresh lung samples were weighed immediately to obtain the wet lung weight and then dehydrated in an 80°C oven for 48 h to obtain the dry lung weight. The W/D ratio was calculated as a reflection of pulmonary edema [26].

### 2.5. Arterial Blood Gas Analysis

The right common carotid artery was isolated and cannulated, and blood samples were collected using a heparinized polyethylene catheter (PE10, Clay Adams; Parsippany, NJ, USA). Partial pressure of oxygen (PaO_2_), partial pressure of carbon dioxide (PaCO_2_), and sodium bicarbonate (HCO_3_^−^) were determined using an automatic blood gas analyzer [27].

### 2.6. Cytokine Detection

The levels of inflammatory cytokines in the lungs, BALFs, or cell culture supernatants were detected by enzyme-linked immunosorbent assay (ELISA). IL-6, tumor necrosis factor-*α* (TNF-*α*), IL-10, IL-1*β*, and IL-18 levels were determined using commercial ELISA kits (Abcam, UK) following the manufacturer's instructions.

### 2.7. Evans Blue Dye (EBD) Extravasation Assay

EBD leakage was used to evaluate pulmonary injury as previously described [28]. In brief, mice were intraperitoneally injected with EBD (30 mg/kg; Sigma, USA), which was then allowed to circulate for an additional 2 h. Then, the lung tissue was perfused via the right ventricle with PBS containing 5 mmol/L EDTA-2Na to remove the intravascular dye from the lung. Lung tissue was then collected and homogenized in formamide for 16 h at 60°C, and the Evans blue absorbance was measured spectrophotometrically at 620 nm.

### 2.8. Western Blot

Total proteins in whole cell lysate (WCL) were extracted from fresh lungs or cells using RIPA lysis buffer and quantified using a BCA protein assay kit [29–31]. Nuclear extracts (NE) were fractionated with NE-PER™ Nuclear and Cytoplasmic Extraction Reagents (Thermo Fisher Scientific, USA) [32]. Samples were then separated on SDS-PAGE gels and transferred to PVDF membranes. Next, the membranes were blocked with 5% BSA at room temperature for 1.5 h, incubated with primary antibodies overnight at 4°C, and then incubated with peroxidase-conjugated secondary antibodies for an additional 1 h at room temperature. Protein bands were then scanned and analyzed using Image Lab software (Bio-Rad, USA). The following primary antibodies were used: anti-p-p65 (#ab76302, Abcam), anti-t-p65 (#ab16502, Abcam), antiproliferating cell nuclear antigen (PCNA; #ab29, Abcam), antinuclear factor E2-related factor 2 (NRF2; #ab62352, Abcam), antiglyceraldehyde-3-phosphate dehydrogenase (GAPDH; #2118S, CST), antiapoptosis-associated speck-like protein (ASC; #ab47092, Abcam), anti-NLRP3 (#ab214185, Abcam), anti-capase-1 p10 (#sc-56036, Santa Cruz), anti-TXNIP (#ab188865, Abcam), anti-p-ASK1 (#3765, CST), anti-t-ASK1 (#8662, CST), anti-p-p38 (#4511, CST), anti-t-p38 (#8690, CST), and antiheat shock protein 20 (HSP20; #ab184161, Abcam).

### 2.9. Real-Time Quantitative Polymerase Chain Reaction

Total RNA was extracted using TRIzol reagent and then reverse transcribed to cDNA using standard protocols [33–35]. Next, the samples were incubated with SYBR Green Mix on a deep-well Real-Time PCR Detection System. Melting curve analysis was performed to examine primer specificity, and relative gene expression was determined using the 2^-*ΔΔ*Ct^ method.

### 2.10. Detection of Lactate Dehydrogenase (LDH), Nuclear Factor-*κ*B (NF-*κ*B), Myeloperoxidase (MPO), Caspase-1, and ASK1 Activity

LDH activity in the lungs and BALFs was determined using an LDH assay kit (Abcam, UK) [36]. Nuclear proteins were extracted, and NF-*κ*B activity was determined using the TransAM kit according to the manufacturer's instructions (Active Motif, USA). MPO activity (Abcam, UK) in fresh lung homogenates was detected by measuring absorbance at 412 nm; this was used as a biomarker for neutrophil infiltration. Caspase-1 activity (Abcam, UK) in the lungs or macrophages was detected using the fluorescent substrate YVAD-AFC and quantified by a multidetection reader. ASK1 activity was measured using an immune complex kinase assay with a His-MKK6 substrate as previously described [37].

### 2.11. Intracellular ROS, Hydrogen Peroxide (H_2_O_2_), and Superoxide Measurements

Intracellular ROS levels were measured in the lungs or macrophages using 2′,7′-dichlorofluorescin diacetate (DCFH-DA; Sigma, USA), which is oxidized to form fluorescent DCF products by excessive free radicals [38–42]. Briefly, the lung homogenates or cells were incubated with DCFH-DA (20 *μ*mol/L) for 1 h at 37°C in the dark, and then DCF fluorescence intensity was detected by a multidetection reader at an excitation/emission wavelength of 485/535 nm. The levels of H_2_O_2_ in the lungs or macrophages were determined by the Amplex™ Red Hydrogen Peroxide/Peroxidase Assay Kit (Thermo Fisher Scientific, USA) according to the manufacturer's instructions [43, 44]. The absorbance was measured using a spectrophotometer at 560 nm. Superoxide production was quantified based on the oxidation of luminol by superoxide as previously described [45]. Samples were prepared and incubated with lucigenin (5 mmol/L; Sigma, USA) for 10 min at 37°C in the dark, and the luminescence intensity was measured at 30 sec intervals for 3-5 min.

### 2.12. Determination of the Levels of Oxidative Products

The levels of protein carbonyls (PCs) were measured using a commercial Protein Carbonyl Content Assay Kit (Abcam, USA) as previously described [6]. In brief, fresh lung samples were homogenized, treated with streptozocin to remove nucleic acids, and incubated with DNPH (100 *μ*L), TCA (30 *μ*L), cold acetone (500 *μ*L), and guanidine solution (200 *μ*L) following the manufacturer's instructions. Then, PCs were measured spectrophotometrically at 375 nm and expressed as pmol/mg protein. The contents of 3-nitrotyrosine (3-NT), malondialdehyde (MDA), and 4-hydroxynonenal (4-HNE) were also detected to assess protein and lipid peroxidation using commercial kits (Abcam, USA). 8-hydroxy 2 deoxyguanosine (8-OHdG) is produced by oxidative DNA damage, and 8-OHdG levels in fresh lung homogenates were evaluated using an 8-OHdG-coated plate and an HRP-conjugated antibody according to the manufacturer's instructions (Abcam, USA). The absorbance was measured at 450 nm and used to calculate total protein concentrations as previously described.

### 2.13. Evaluation of DNA Fragmentation, Total Antioxidant Capacity (TAOC), Total Superoxide Dismutase (SOD), Catalase (CAT), and NRF2 Activity

Cytoplasmic histone-associated DNA fragments were detected to further confirm DNA damage using a commercial cell death detection ELISA (Roche Applied Science, USA) at 405 nm as previously described [46]. TAOC, total SOD, and CAT activities in the lungs or macrophages were measured by commercial kits according to the manufacturer's instructions (Abcam, USA). To evaluate NRF2 transcription activity, nuclear extracts were prepared and incubated with the TransAM NRF2 ELISA kit (Active Motif, USA) and then spectrophotometrically detected at 450 nm.

### 2.14. Murine Peritoneal Macrophage Isolation and Treatment

Primary murine macrophages were isolated from the peritoneal cavity via lavage. Cells were centrifuged at 1500 g for 10 min at 4°C, resuspended in RPMI 1640 medium, and seeded in 6-well plates at a density of 2 × 10^6^ cells/well [7, 26]. The cells were transfected with miR-23a-5p antagomir (50 nmol/L), agomir (50 nmol/L), or the negative controls using Lipofectamine™ RNAiMAX Transfection Reagent (Thermo Fisher Scientific, USA). Cells were incubated in transfection medium for 24 h, incubated in fresh medium for an additional 24 h, and then stimulated with LPS (100 ng/mL) for 6 h [47]. To silence ASK1, macrophages were infected for 6 h with a lentiviral vector carrying either a short hairpin RNA targeting ASK1 (shASK1) or a scramble RNA (shScramble) at a multiplicity of infection of 50. Cells were then incubated in fresh medium for an additional 24 h prior to miR-23a-5p overexpression. The mouse ASK1 targeting sequences were obtained from Santa Cruz and then packaged into lentiviral vectors by Vigene Bioscience (Rockville, USA).

### 2.15. Luciferase Reporter Assay

HEK293T cells were seeded in 24-well plates at a density of 1 × 10^5^ cells/well. 48 h later, cells were cotransfected with a pGL3 plasmid (Promega, USA) carrying either the wild type (WT) or mutant (MUT) HSP20 3′ UTR with or without miR-23a-5p agomir using Lipofectamine™ RNAiMAX Transfection Reagent [48–50]. The cells were incubated for 48 h, and then luciferase activities were measured using a luciferase reporter assay kit (Promega, USA).

### 2.16. Statistical Analysis

All data are presented as the means ± SD and were analyzed using SPSS 23.0 software (SPSS Inc., USA). Differences between two groups were compared by an unpaired two-sided Student's *t*-test. For multigroup comparisons, one-way ANOVA followed by the Newman–Keuls post hoc test was performed. A *P* value less than 0.05 was considered statistically significant.

## 3. Results

### 3.1. miR-23a-5p Antagomir Ameliorates Pulmonary Injury and Dysfunction in LPS-Treated Mice

**Figure 1:**
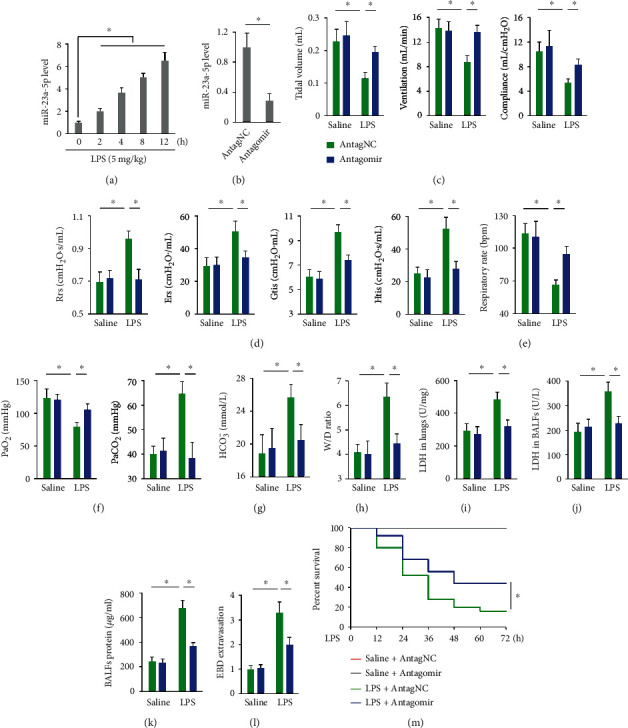
miR-23a-5p antagomir ameliorates pulmonary injury and dysfunction in LPS-treated mice. (a) Mice were intratracheally treated with LPS (5 mg/kg), and the level of miR-23a-5p in the lungs was measured at indicating times. (b) Mice were treated with the miR-23a-5p antagomir (80 mg/kg/day) or AntagNC for 3 consecutive days through tail-vein injections, and then the level of miR-23a-5p in the lungs was measured. (c–e) Mice were pretreated with the miR-23a-5p antagomir (80 mg/kg/day) or AntagNC for 3 consecutive days and then intratracheally injected with 5 mg/kg LPS. 12 h after LPS injection, the mice received pulmonary function measurements. Respiratory system resistance (Rrs), elastance (Ers), tissue damping (Gtis), and tissue elastance (Htis) belong to the invasive pulmonary functional parameters, as determined using the forced oscillation technique and the constant-phase model. (f and g) Arterial blood gas analysis of PaO_2_, PaCO_2_, and HCO_3_^−^. (h) Lung W/D ration in mice. (i and j) LDH activities in the lungs and BALFs. (k) Total protein concentrations in BALFs. (l) EBD extravasation to the lungs. (m) Mice were pretreated with the miR-23a-5p antagomir or AntagNC for 3 consecutive days and then exposed to a lethal dose of LPS (25 mg/kg). Mice were observed every 12 h over 72 h with the percent survival calculated. The data are expressed as the means ± SD (*n* = 6 per group). ^∗^*P* < 0.05 when compared with the matched group.

We first investigated whether miR-23a-5p expression was altered during ALI and detected that miR-23a-5p levels were increased in the lungs from LPS-treated mice (Figure 1(a)). We then used miR-23a-5p antagomir to inhibit miR-23a-5p expression in vivo, and the efficiency was confirmed in Figure 1(b). As shown in Figure 1(c), LPS injection significantly decreased tidal volume, pulmonary ventilation, and lung compliance of mice that were prevented by the miR-23a-5p antagomir. Compared with saline-treated mice, the mice treated with LPS displayed higher Rrs, Ers, Gtis, and Htis values, while miR-23a-5p antagomir administration improved all measures of lung function (Figure 1(d)) In addition, the miR-23a-5p antagomir also restored respiratory rates in LPS-treated mice (Figure 1(e)). In line with their compromised pulmonary function, LPS-treated mice displayed decreased PaO_2_ and increased PaCO_2_ and HCO_3_^−^ levels, which were attenuated upon miR-23a-5p antagomir administration (Figures 1(f) and 1(g)). LPS induced severe pulmonary edema and damage in control mice, yet to a less extent in miR-23a-5p antagomir-treated mice, as evidenced by decreased W/D ratio and LDH activities in the lungs or BALFs (Figures 1(h)–1(j)). The levels of BALFs proteins and EBD extravasation further clarified the protective role of the miR-23a-5p antagomir against LPS-induced ALI (Figures 1(k) and 1(l)). Moreover, we found that treatment with the miR-23a-5p antagomir evidently improved the survival rates of LPS-challenged mice (Figure 1(m)). These findings demonstrate that miR-23a-5p is increased in LPS-injured lungs and that pharmacological inhibition of miR-23a-5p remarkably ameliorates LPS-induced pulmonary injury and dysfunction in mice.

### 3.2. miR-23a-5p Agomir Exacerbates LPS-Induced ALI in Mice

**Figure 2:**
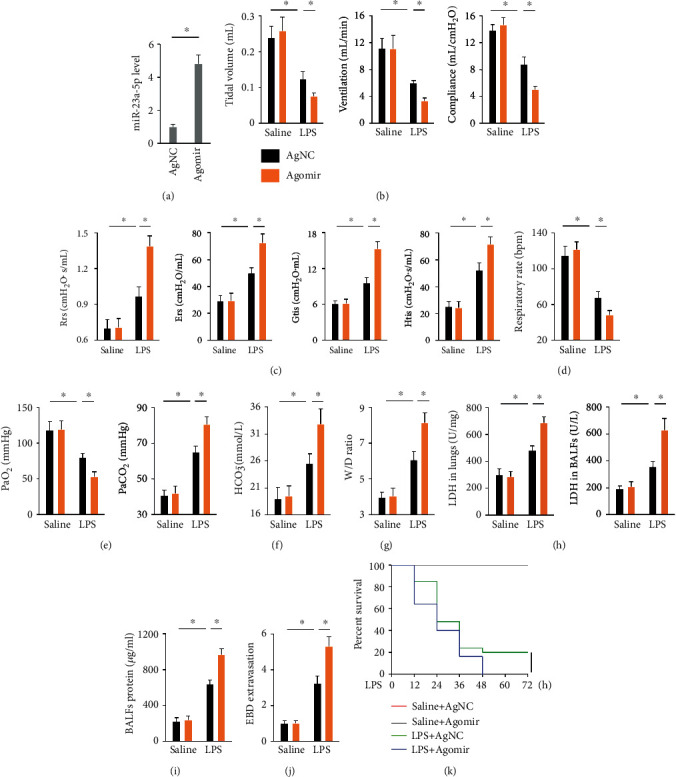
miR-23a-5p agomir exacerbates LPS-induced ALI in mice. (a) Mice were treated with miR-23a-5p agomir (30 mg/kg/day) or AgNC for 3 consecutive days through tail-vein injections, and then the level of miR-23a-5p in the lungs was measured. (b–d) Mice were treated with the miR-23a-5p agomir (30 mg/kg/day) or AgNC for 3 consecutive days and then intratracheally injected with 5 mg/kg LPS. 12 h after LPS injection, the mice received pulmonary function measurements. (e and f) Arterial blood gas analysis of PaO_2_, PaCO_2_, and HCO_3_^−^. (g) Lung W/D ration in mice. (h) LDH activities in the lungs and BALFs. (i) Total protein concentrations in BALFs. (j) EBD extravasation to the lungs. (k) Mice were pretreated with the miR-23a-5p agomir or AgNC for 3 consecutive days and then exposed to a lethal dose of LPS (25 mg/kg). Mice were observed every 12 h over 72 h with the percent survival calculated. The data are expressed as the means ± SD (*n* = 6 per group). ^∗^*P* < 0.05 when compared with the matched group.

To examine whether miR-23a-5p upregulation would exacerbate LPS-induced ALI, mice were treated with the miR-23a-5p agomir to elevate pulmonary miR-23a-5p expression (Figure 2(a)). As shown in Figures 2(b)–2(d), the miR-23a-5p agomir further decreased tidal volume, pulmonary ventilation, lung compliance, and respiratory rates and increased Rrs, Ers, Gtis, and Htis values of LPS-treated mice. Consistently, mice treated with the miR-23a-5p agomir displayed increased gas exchange impairment following LPS injection, as indicated by the decreased PaO_2_ and increased PaCO_2_ and HCO_3_^−^ levels (Figures 2(e) and 2(f)). Besides, the miR-23a-5p agomir exacerbated LPS-related pulmonary edema, cellular injury, and structural destruction in mice (Figures 2(g)–2(j)). Furthermore, mice treated with the miR-23a-5p agomir had lower survival rates after LPS stimulation (Figure 2(k)). Taken together, these results show that the miR-23a-5p agomir exacerbates LPS-induced ALI in mice.

### 3.3. miR-23a-5p Antagomir Inhibits the Inflammatory Response in ALI Mice

**Figure 3:**
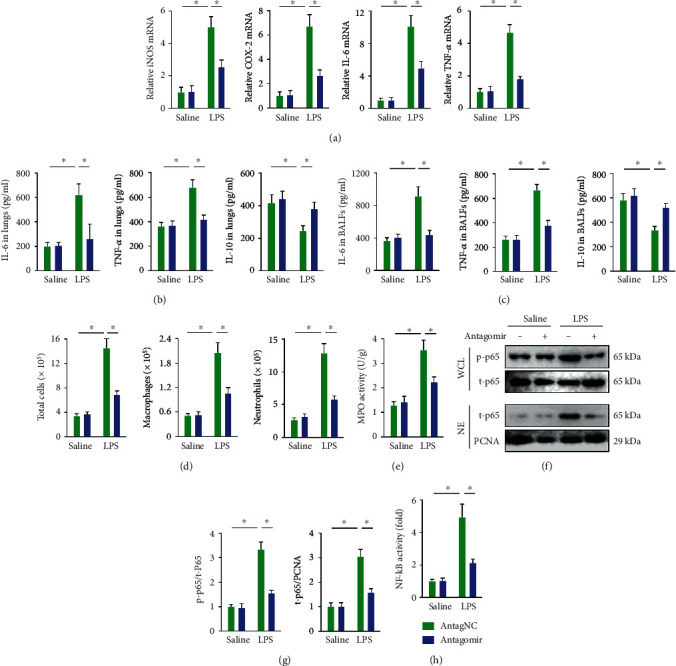
miR-23a-5p antagomir inhibits the inflammatory response in ALI mice. (a) Relative mRNA levels of inflammatory markers in the lungs with or without the miR-23a-5p antagomir treatment upon LPS injection. (b and c) The levels of inflammatory markers in the lungs or BALFs. (d) Total cells, macrophages, and neutrophils in BALFs were determined. (e) MPO activity in the lungs. (f and g) The phosphorylation and nuclear accumulation of p65 were detected by western blot. (h) Relative NF-*κ*B activity in the lungs. The data are expressed as the means ± SD (*n* = 6 per group). ^∗^*P* < 0.05 when compared with the matched group.

Next, we detected the effects of the miR-23a-5p antagomir on the intrapulmonary inflammatory response in ALI mice. We found that miR-23a-5p antagomir treatment effectively reduced inflammation-associated genes expression in the lungs, including inducible nitric oxide synthase (iNOS, also known as NOS2), cyclooxygenase-2 (COX-2), IL-6, and TNF-*α* (Figure 3(a)). The miR-23a-5p antagomir also decreased the levels of proinflammatory cytokines (IL-6 and TNF-*α*) and increased the levels of anti-inflammatory cytokines (IL-10) in the lungs and BALFs (Figures 3(b) and 3(c)). Besides, the miR-23a-5p antagomir suppressed the accumulation of total cells, macrophages, and neutrophils in BALFs following LPS injection, which was further confirmed by the decreased MPO activity in murine lungs (Figures 3(d) and 3(e)). NF-*κ*B is the most critical transcription factor involved in the inflammatory response and is mainly sequestered in the cytoplasm under physiological conditions. Upon LPS stimulation, it translocates to the nucleus to trigger the expression of multiple inflammatory cytokines [51, 52]. Herein, we observed that the miR-23a-5p antagomir inhibited the phosphorylation and nuclear accumulation of p65 in LPS-injured lungs (Figures 3(f) and 3(g)). Accordingly, the LPS-induced increase in NF-*κ*B activity was also suppressed by the miR-23a-5p antagomir (Figure 3(h)). Collectively, these data indicate that the miR-23a-5p antagomir inhibits the inflammatory response in ALI mice.

### 3.4. miR-23a-5p Antagomir Decreases Oxidative Stress in ALI Mice

**Figure 4:**
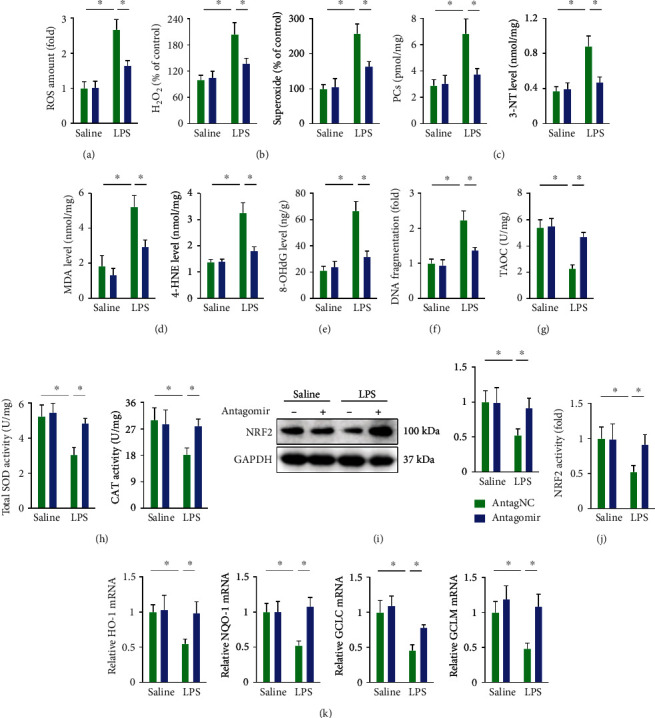
miR-23a-5p antagomir decreases oxidative stress in ALI mice. (a) Intracellular ROS amount. (b) Relative levels of H_2_O_2_ and superoxide in the lungs. (c) Oxidative products from proteins in the lungs. (d) Oxidative products from lipids in the lungs. (e) Oxidative products from DNA in the lungs. (f) Relative level of DNA fragmentation in the lungs. (g and h) Cellular antioxidant capacity is determined by TAOC, total SOD, and CAT activities. (i) NRF2 protein levels. (j and k) Relative NRF2 activity and mRNA levels of the downstream targets. The data are expressed as the means ± SD (*n* = 6 per group). ^∗^*P* < 0.05 when compared with the matched group.

Oxidative damage also contributes to the progression of LPS-induced ALI; therefore, we examined whether the miR-23a-5p antagomir could inhibit LPS-induced oxidative stress in the lungs [26, 45]. As shown in Figure 4(a), ROS generation was significantly increased in the lungs of ALI mice, but was suppressed by miR-23a-5p antagomir treatment. H_2_O_2_ and superoxide are two primary forms of ROS that play vital roles in LPS-induced oxidative damage to the lungs. Intriguingly, the miR-23a-5p antagomir significantly decreased H_2_O_2_ and superoxide levels in LPS-treated lungs (Figure 4(b)). ROS overproduction induces oxidative damage to biomacromolecules and elevates the levels of oxidative products from protein (e.g., PCs and 3-NT), lipid (e.g., MDA and 4-HNE), and DNA (e.g., 8-OHdG). As expected, LPS injection increased the levels of PCs, 3-NT, MDA, 4-HNE, and 8-OHdG in the lungs, which were significantly decreased by the miR-23a-5p antagomir (Figures 4(c)–4(e)). Besides, the miR-23a-5p antagomir also reduced LPS-induced DNA damage, as evidenced by lower DNA fragmentation levels (Figure 4(f)). Cellular antioxidant capacity confers protective effects against LPS-induced ALI; however, our data found that TAOC and the antioxidant enzymes, SOD, and CAT activities were significantly suppressed in the lungs of ALI mice. Fortunately, the miR-23a-5p antagomir restored the antioxidant capacity in LPS-injured lungs (Figures 4(g) and 4(h)). Due to the pivotal role of NRF2 in regulating the expression of numerous antioxidant enzymes, we investigated whether the miR-23a-5p antagomir could affect the NRF2 pathway. As shown in Figure 4(i), the decreased NRF2 protein level in LPS-treated lungs was prevented by the miR-23a-5p antagomir. Moreover, the miR-23a-5p antagomir also preserved NRF2 transcription activity upon LPS stimulation; this was further confirmed by the increased mRNA levels of downstream targets, such as heme oxygenase-1 (HO-1), NAD(P)H quinone dehydrogenase 1 (NQO1), glutamate-cysteine ligase catalytic subunit (GCLC), and glutamate-cysteine ligase modifier subunit (GCLM) (Figures 4(j) and 4(k)). These results suggest that the miR-23a-5p antagomir decreases oxidative stress in ALI mice.

### 3.5. miR-23a-5p Antagomir Suppresses NLRP3 Inflammasome Activation in ALI Mice

**Figure 5:**
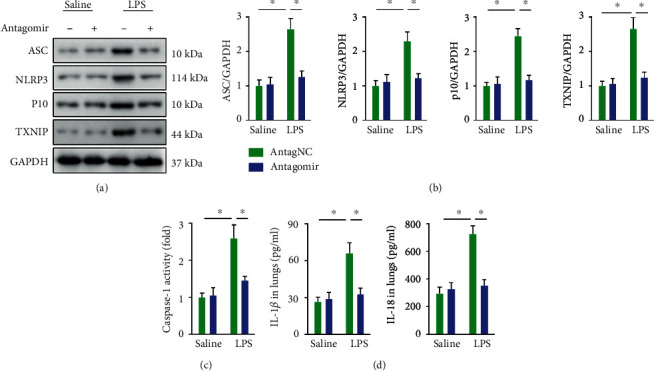
miR-23a-5p antagomir suppresses NLRP3 inflammasome activation in ALI mice. (a and b) ASC, NLRP3, p10, and TXNIP protein levels. (c) Relative caspase-1 activity in the lungs. (d) The levels of IL-1*β* and IL-18 in the lungs. The data are expressed as the means ± SD (*n* = 6 per group). ^∗^*P* < 0.05 when compared with the matched group.

Increased ROS promotes the disassociation of TXNIP from thioredoxin and activates the NLRP3 inflammasome, which in turn amplifies the inflammatory response via accelerating the maturation and release of proinflammatory cytokines [53–55]. Herein, we found that the LPS challenge elevated the protein abundances of ASC, NLRP3, TXNIP, and the active form of caspase-1 (p10), whereas these alterations were remarkably blunted by the miR-23a-5p antagomir (Figures 5(a) and 5(b)). The miR-23a-5p antagomir also suppressed caspase-1 activity and reduced the levels of IL-1*β* and IL-18 in the lungs (Figures 5(c) and 5(d)). These data imply that the miR-23a-5p antagomir suppresses NLRP3 inflammasome activation in ALI mice.

### 3.6. miR-23a-5p Agomir Aggravates Pulmonary Inflammation in ALI Mice

In contrast, the miR-23a-5p agomir significantly promoted the accumulation of leukocytes in BALFs upon LPS stimulation; this was further verified by the increased pulmonary MPO activity (Figures S1A-B). Besides, LPS-induced increases in IL-6, TNF-*α*, and decrease of IL-10 in BALFs or lungs were more pronounced following treatment with the miR-23a-5p agomir (Figures S1C-D). As expected, NF-*κ*B transcription activity was further enhanced in the miR-23a-5p agomir-treated mice upon LPS injection (Figure S1E). These results demonstrate that the miR-23a-5p agomir aggravates pulmonary inflammation in ALI mice.

### 3.7. miR-23a-5p Agomir Increases Pulmonary Oxidative Damage and NLRP3 Inflammasome Activation in ALI Mice

The miR-23a-5p agomir also elevated pulmonary ROS, H_2_O_2_, and superoxide levels in LPS-treated mice (Figures S2A-B). Accordingly, LPS-associated generations of the oxidative products from protein, lipid, and DNA were further increased in mice treated with the miR-23a-5p agomir (Figures S2C-G). Caspase-1 activation and IL-1*β* and IL-18 overproduction were also augmented by the miR-23a-5p agomir (Figures S2H-I). These findings show that the miR-23a-5p agomir increases pulmonary oxidative damage and NLRP3 inflammasome activation in ALI mice.

### 3.8. miR-23a-5p Antagomir Blocks LPS-Induced Inflammation and Oxidative Stress in Macrophages

**Figure 6:**
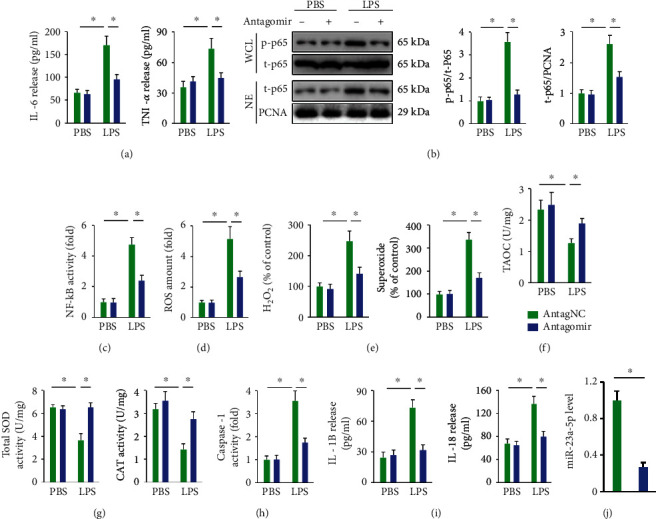
miR-23a-5p antagomir blocks LPS-induced inflammation and oxidative stress in macrophages. (a) Primary macrophages were incubated with the miR-23a-5p antagomir (50 nmol/L) or AntagNC for 24 h and then incubated in fresh medium for additional 24 h before LPS (100 ng/mL) stimulation for 6 h. The levels of IL-6 and TNF-*α* in the culture supernatants from LPS-treated macrophages were determined. (b) The phosphorylation and nuclear accumulation of p65 were detected by western blot. (c) Relative NF-*κ*B activity in macrophages. (d) Intracellular ROS amount. (e) Relative levels of H_2_O_2_ and superoxide in macrophages. (f and g) Cellular antioxidant capacity is determined by TAOC, total SOD, and CAT activities. (h) Relative caspase-1 activity in macrophages. (i) The levels of IL-1*β* and IL-18 in the culture supernatants from LPS-treated macrophages. (j) Relative miR-23a-5p levels in primary macrophages with or without the miR-23a-5p antagomir treatment. The data are expressed as the means ± SD (*n* = 6 per group). ^∗^*P* < 0.05 when compared with the matched group.

Based on the in vivo findings, we then explored whether the miR-23a-5p antagomir could block LPS-induced inflammation in primary macrophages in vitro. Consistent with the in vivo data, the miR-23a-5p antagomir notably decreased IL-6 and TNF-*α* releases from LPS-treated macrophages (Figure 6(a)). Besides, p65 phosphorylation, nuclear accumulation, and NF-*κ*B activity were also inhibited by miR-23a-5p antagomir treatment in LPS-stimulated macrophages (Figures 6(b) and 6(c)). As shown in Figures 6(d) and 6(e), primary macrophages treated with the miR-23a-5p antagomir also showed lower levels of intracellular ROS, H_2_O_2_, and superoxide compared to those in the LPS group. Meanwhile, antioxidant capacity was preserved by the miR-23a-5p antagomir in LPS-treated macrophages (Figures 6(f) and 6(g)). We also found that the miR-23a-5p antagomir markedly inhibited LPS-induced activation of caspase-1 and the releases of IL-1*β* and IL-18 in primary macrophages (Figures 6(h) and 6(i)). The efficiency of the miR-23a-5p antagomir was verified in Figure 6(j). Together, these results suggest that the miR-23a-5p antagomir blocks LPS-induced inflammation and oxidative stress in macrophages.

### 3.9. miR-23a-5p Agomir Promotes LPS-Induced Inflammation and Oxidative Stress in Macrophages

We also treated macrophages with the miR-23a-5p agomir to determine whether increased miR-23a-5p would aggravate inflammation and oxidative stress in LPS-stimulated macrophages (Figure S3A). As expected, the miR-23a-5p agomir further promoted the releases of proinflammatory cytokines and the generation of free radicals from LPS-treated primary macrophages (Figures S3B-D). Endogenous antioxidant capacity was lower LPS-stimulated macrophages treated with the miR-23a-5p agomir than in those treated with the control agomir (Figures S3E-F). Caspase-1 activation and the releases of IL-1*β* and IL-18 from LPS-treated macrophages were further augmented by the miR-23a-5p agomir (Figures S3G-H). These findings strongly indicate that the miR-23a-5p agomir promotes LPS-induced inflammation and oxidative stress in macrophages.

### 3.10. mir-23a-5p Agomir Augments LPS-Induced Inflammation and Oxidative Stress via Activating ASK1 In Vitro

**Figure 7:**
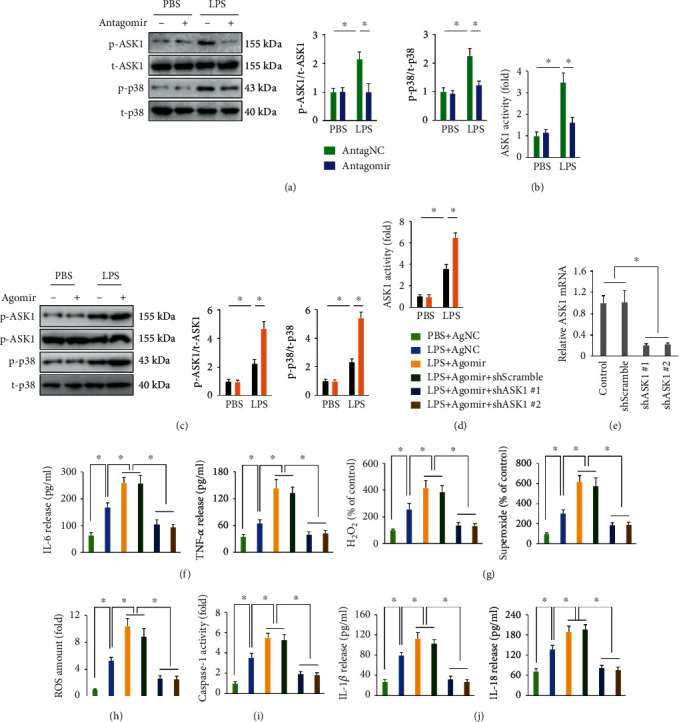
miR-23a-5p agomir augments LPS-induced inflammation and oxidative stress via activating ASK1 in vitro. (a and b) Relative ASK1 phosphorylation and activity in macrophages with or without miR-23a-5p antagomir treatment upon LPS stimulation. (c and d) Relative ASK1 phosphorylation and activity in macrophages with or without the miR-23a-5p agomir treatment upon LPS stimulation. (e) Relative ASK1 mRNA level in macrophages. (f) Primary macrophages were incubated with the miR-23a-5p antagomir (50 nmol/L) or AntagNC for 24 h and then incubated in fresh medium for additional 24 h before LPS (100 ng/mL) stimulation for 6 h. To silence ASK1, macrophages were infected with shASK1 or shScramble at a multiplicity of infection of 50 for 6 h, which were then incubated in fresh medium for additional 24 hours before miR-23a-5p agomir treatment. The levels of IL-6 and TNF-*α* in the culture supernatants were measured. (g) Relative levels of H_2_O_2_ and superoxide in macrophages. (h) Intracellular ROS amount. (i) Relative caspase-1 activity in macrophages. (j) The levels of IL-1*β* and IL-18 in the culture supernatants from LPS-treated macrophages. The data are expressed as the means ± SD (*n* = 6 per group). ^∗^*P* < 0.05 when compared with the matched group.

We then investigated whether ASK1 was involved in the deleterious effects of the miR-23a-5p agomir in LPS-stimulated macrophages. As shown in Figures 7(a) and 7(b), the miR-23a-5p antagomir inhibited both ASK1 phosphorylation and ASK1 activity in LPS-treated macrophages; this was further evidenced by decreased phosphorylation of the downstream p38 kinase. Conversely, the miR-23a-5p agomir increased ASK1 phosphorylation and activity in LPS-treated macrophages (Figures 7(c) and 7(d)). Next, ASK1 in macrophages was knocked down by two different shASK1 vectors, and the efficiency was verified in Figure 7(e). As shown in Figures 7(f)–7(h), ASK1 silence decreased the levels of proinflammatory cytokines and oxidative stress in miR-23a-5p agomir-treated macrophages upon LPS stimulation. Besides, caspase-1 activation and the increased releases of IL-1*β* and IL-18 from LPS-stimulated macrophages were also blocked by ASK1 silence (Figures 7(i) and 7(j)). These results strongly indicate that the miR-23a-5p agomir augments LPS-induced inflammation and oxidative stress via activating ASK1 in vitro.

### 3.11. ASK1 Inhibition Abrogates the Deleterious Effects of the miR-23a-5p Agomir In Vivo

**Figure 8:**
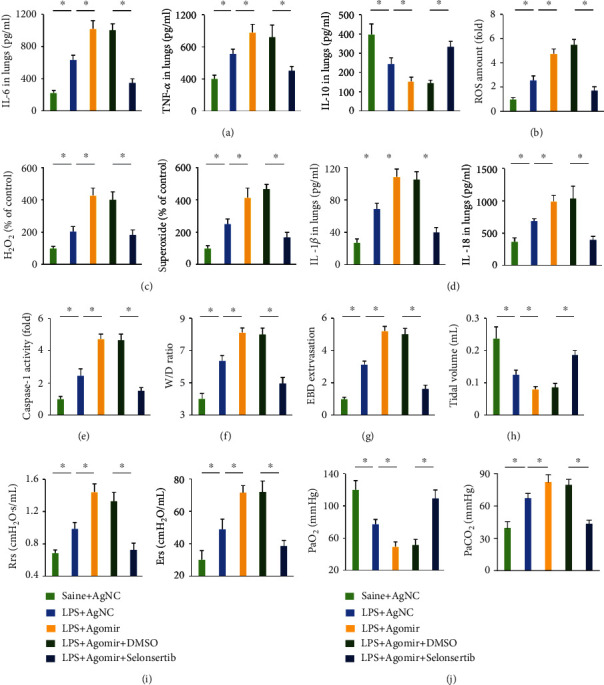
ASK1 inhibition abrogates the deleterious effects of miR-23a-5p agomir in vivo. (a) Mice were treated with the miR-23a-5p agomir (30 mg/kg/day) or AgNC for 3 consecutive days and then intratracheally injected with 5 mg/kg LPS. To inhibit endogenous ASK1, mice were daily treated with selonsertib (4 mg/kg; Selleck, USA) for 7 consecutive days prior LPS injection. IL-6 and TNF-*α* levels in the lungs were measured. (b) Intracellular ROS amount. (c) Relative levels of H_2_O_2_ and superoxide in the lungs. (d) Relative caspase-1 activity in the lungs. (e) The levels of IL-1*β* and IL-18 in the lungs. (f) Lung W/D ration in mice. (g) EBD extravasation to the lungs. (h and i) Pulmonary functional parameters. (j) Arterial blood gas analysis of PaO_2_ and PaCO_2_. The data are expressed as the means ± SD (*n* = 6 per group). ^∗^*P* < 0.05 when compared with the matched group.

Selonsertib, a potent ASK1 inhibitor, was then used to suppress ASK1 activity in mice [22]. Consistent with the in vitro findings, selonsertib significantly reduced the levels of IL-6 and TNF-*α*, while elevated IL-10 expression in the lungs (Figure 8(a)). Besides, miR-23a-5p agomir-associated increases in intracellular ROS, H_2_O_2_, and superoxide in response to LPS injection were decreased by selonsertib treatment (Figures 8(b) and 8(c)). NLRP3 activation was also blocked by ASK1 inhibition, as evidenced by decreased levels of IL-1*β*, IL-18, and caspase-1 activity (Figures 8(d) and 8(e)). Due to the alleviation of inflammation and oxidative stress, mice treated with selonsertib also displayed reduced pulmonary edema and injury (Figures 8(f) and 8(g)). The miR-23a-5p agomir-induced impairment of pulmonary function and gas exchange was also partially restored by selonsertib, as confirmed by the increased tidal volume, PaO_2_, and decreased Rrs, Ers, and PaCO_2_ of LPS-treated mice (Figures 8(h)–8(j)). These data demonstrate that ASK1 inhibition abrogates the deleterious effects of the miR-23a-5p agomir in vivo.

### 3.12. miR-23a-5p Agomir Activates ASK1 via Directly Reducing HSP20 Expression

**Figure 9:**
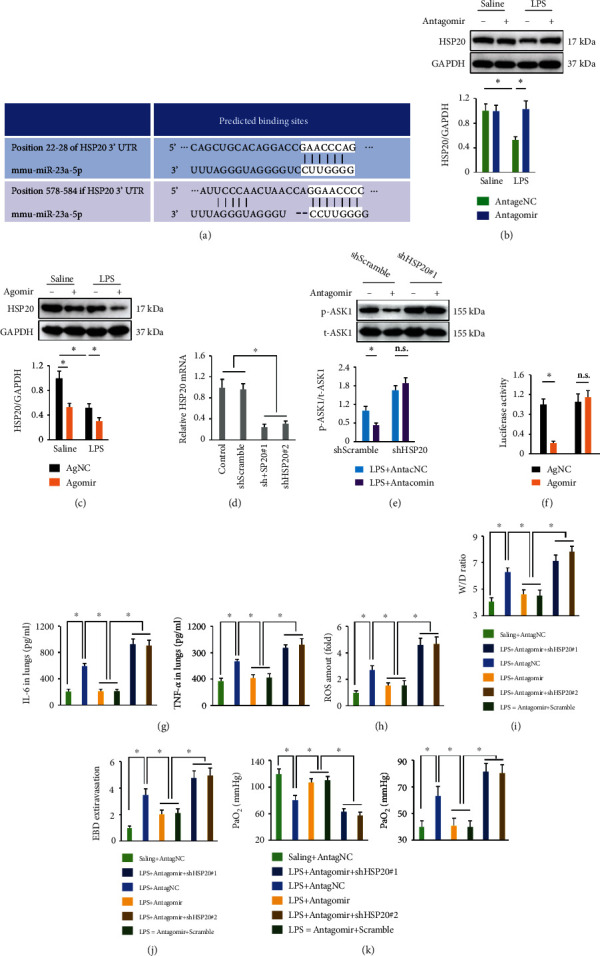
miR-23a-5p agomir activates ASK1 via directly reducing HSP20 expression. (a) The predicted miR-23a-5p-binding sites within the 3′ UTR of HSP20. (b and c) HSP20 protein levels in the lungs with miR-23a-5p antagomir or agomir treatment upon LPS injection. (d) Relative HSP20 mRNA level in the lungs. (e) Mice were pretreated with the miR-23a-5p antagomir (80 mg/kg/day) or AntagNC for 3 consecutive days and then intratracheally injected with 5 mg/kg LPS. To knock down endogenous HSP20, mice were intratracheally injected with shHSP20 (1 × 10^8^ PFU per mouse) or shScramble 1 week before LPS treatment. ASK1 phosphorylation was detected by western blot. (f) Relative luciferase activity. (g) IL-6 and TNF-*α* levels in the lungs were measured. (h) Intracellular ROS amount. (i) Lung W/D ration in mice. (j) EBD extravasation to the lungs. (k) Arterial blood gas analysis of PaO_2_ and PaCO_2_. The data are expressed as the means ± SD (*n* = 6 per group). ^∗^*P* < 0.05 when compared with the matched group, n.s. indicates no significance.

Previous studies have reported that HSP20 is required for ASK1 inhibition and that HSP20 protects against LPS-induced organic injury, including ALI [8, 9, 56]. Herein, we identified two potential binding sites in the 3′ UTR of HSP20 using the online TargetScan software (Figure 9(a)). Besides, we observed that the miR-23a-5p antagomir preserved HSP20 protein levels in LPS-treated lungs (Figure 9(b)). While the miR-23a-5p agomir suppressed HSP20 in the lungs with or without LPS injury (Figure 9(c)). To clarify the involvement of HSP20, we knocked down HSP20 expression in murine lungs and the efficiency was verified in Figure 9(d). As shown in Figure 9(e), the miR-23a-5p antagomir significantly suppressed ASK1 phosphorylation in shScramble-infected mice, yet failed to do so after HSP20 silence in response to LPS injection. A luciferase reporter assay demonstrated that the miR-23a-5p agomir inhibited luciferase activity in cells transfected with the WT HSP20 3′ UTR, but did not alter luciferase activity in cells transfected with the MUT HSP20 3′ UTR (Figure 9(f)). These results clearly demonstrated that miR-23a-5p directly binds to the HSP20 3′ UTR. We found that HSP20 silence abrogated the anti-inflammatory and antioxidant effects of the miR-23a-5p antagomir in ALI mice, as confirmed by the unaffected IL-6, TNF-*α*, and ROS levels in the lungs (Figures 9(g)–9(h)). Accordingly, the improvements in pulmonary edema and injury and gas exchange were blocked upon HSP20 knockdown (Figures 9(i)–9(k)). These findings indicate that the miR-23a-5p agomir activates ASK1 via directly reducing HSP20 expression.

## 4. Discussion

**Figure 10:**
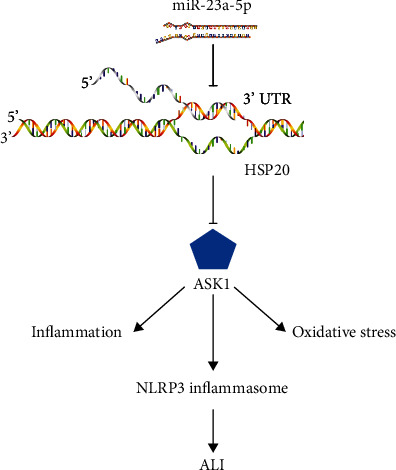
Schematic model of the role of miR-23a-5p on HSP20/ASK1 signaling during ALI. miR-23a-5p directly binds to the 3′ UTR of HSP20 and inhibits its protein expression, which then activates ASK1 to augment inflammation and oxidative stress in ALI.

The present study is aimed at investigating the role and potential mechanism of miR-23a-5p in ALI development. For this purpose, our results reveal that miR-23a-5p is upregulated in murine lungs in response to LPS injury, and that miR-23a-5p antagomir significantly prevents LPS-induced ALI in mice via inhibiting inflammation and oxidative stress. Conversely, the miR-23a-5p agomir aggravates the inflammatory response and oxidative damage generated during LPS-induced pulmonary injury. Besides, we also find that the miR-23a-5p antagomir reduces, while the miR-23a-5p agomir promotes LPS-associated proinflammatory cytokine releases and ROS overproduction in primary macrophages. Additionally, the deleterious effects of miR-23a-5p are mediated through ASK1 activation, and these effects can be blunted by pharmacological or genetic suppression of ASK1. Moreover, we determine that miR-23a-5p directly binds to the 3′ UTR of HSP20 and that HSP20 silence abrogates the anti-inflammatory and antioxidant effects of the miR-23a-5p antagomir in ALI mice (Figure 10). Overall, this study demonstrates for the first time the involvement of miR-23a-5p in the regulation of LPS-induced ALI and identifies miR-23a-5p as a potential therapeutic candidate for the treatment of ALI.

Overproduction of the proinflammatory cytokines and free radicals contributes to the initiation and development of LPS-induced ALI [7, 26]. Upon LPS stimulation, normal pulmonary structure and barrier function are disrupted, and leukocytes subsequently migrate to lung tissues, where they in turn recruit more inflammatory cells via producing multiple proinflammatory cytokines [3, 4]. Accordingly, we herein observed that pulmonary barrier function was markedly compromised in ALI mice, as verified by the increased EBD extravasation and leukocyte counts in BALFs; however, miR-23a-5p antagomir treatment could ameliorate these pathological alterations. LPS itself and the accumulated inflammatory cells both contribute to the generation of free radicals that ultimately overwhelm the endogenous antioxidant capacity of the lungs and result in severe oxidative damage to lung cells. In addition, it has been reported that ROS acts as the primary activator of the NLRP3 inflammasome, thereby accelerating the maturation and release of proinflammatory cytokines, including IL-1*β* and IL-18 [26]. In this study, we observed that the miR-23a-5p antagomir preserved the intracellular antioxidant capacity of LPS-treated lungs and suppressed the activation of the NLRP3 inflammasome. Various studies have revealed the indispensability of ASK1 in regulating inflammation and oxidative stress under different pathological stimuli. Hayakawa et al. found that ASK1 deficiency increased susceptibility to colonic inflammation in mice with inflammatory bowel diseases [57]. Data from Qin and colleagues implied that ASK1 activation increased NF-*κ*B activity and inflammatory cytokine/chemokine expressions during hepatic ischemia/reperfusion injury [58]. Besides, ASK1 could enhance NF-*κ*B activity via the downstream p38 kinase, whereas attenuation of the ASK1/p38 pathway remarkably decreased the expression of proinflammatory cytokines [59]. ASK1 deficiency also lessened NADPH oxidase-mediated free radical production and reduced aldosterone-induced cardiac oxidative stress [60]. Moreover, ASK1 is also associated with various lung diseases via the regulation of inflammation and oxidative stress, such as pulmonary arterial hypertension, chronic obstructive pulmonary disease, and ALI [5, 10, 61, 62]. Previous studies have identified HSP20 as an upstream inhibitor of ASK1, and HSP20 overexpression rendered ASK1 inaccessible to activation, resulting in reduced activity of the downstream p38 signaling cascade [8]. In this study, we demonstrated that HSP20/ASK1 was involved in the regulation of LPS-induced ALI by miR-23a-5p. Intriguingly, we found that the miR-23a-5p antagomir conferred a partial, not complete, reversal of some parameters (e.g., iNOS, COX-2, and NF-*κ*B). As we know, multiple complex mechanisms contribute to the pathogenesis of ALI. In the present study, we investigated the possible involvement of miR-23a-5p in LPS-induced ALI from the view of inflammation and oxidative stress. As presented in our study, the miR-23a-5p antagomir also failed to completely restore LPS-induced pulmonary dysfunction. This phenomenon can be ascribed to the existence of alternative pathogenic factors to ALI independent of miR-23a-5p. Of course, the insufficient efficiency of the miR-23a-5p antagomir may also contribute to this result.

In summary, our findings suggest that LPS-induced miR-23a-5p upregulation contributes to the development of pulmonary injury and dysfunction. Inhibition of endogenous miR-23a-5p provides pulmonary protection against LPS-induced ALI. Collectively, our data indicate that miR-23a-5p is a valuable therapeutic candidate for the treatment of ALI.

## Data Availability

The data that support the findings of this study are available from the corresponding author upon reasonable request.

## Supplementary Materials

Supplementary Materials Figure S1: miR-23a-5p agomir aggravates the pulmonary inflammation in ALI mice. Figure S2: miR-23a-5p agomir increases pulmonary oxidative damage and NLRP3 inflammasome activation in ALI mice. Figure S3: miR-23a-5p agomir promotes LPS-induced inflammation and oxidative stress in macrophages.
