# Supplementary material for: MicroRNA-23a-5p Is Involved in the Regulation of Lipopolysaccharide-Induced Acute Lung Injury by Targeting HSP20/ASK1
Source: Oxid Med Cell Longev. 2021 Aug 11;2021:9942557. doi: 10.1155/2021/9942557 (PMC8376430; doi:10.1155/2021/9942557)
Supplement: Supplementary Materials — Figure S1: miR-23a-5p agomir aggravates the pulmonary inflammation in ALI mice. Figure S2: miR-23a-5p agomir increases pulmonary oxidative damage and NLRP3 inflammasome activation in ALI mice. Figure S3: miR-23a-5p agomir promotes LPS-induced inflammation and oxidative stress in macrophages. [file 9942557.f1.docx]

**MicroRNA-23a-5p is involved in the regulation of lipopolysaccharide-induced acute lung injury by targeting HSP20/ASK1**

Yi-Fei Chen^1 *^, Fen Hu^2 *^, Xian-Guo Wang^3^, Zheng Tang^3^, He-Xiao Tang^3^, Ming Xu^3 #^

^1^ Department of Respiratory and Critical Care Medicine, Zhongnan Hospital of Wuhan University, Wuhan 430071, Hubei, China

^2^ Department of Respiratory Medicine, The First People's Hospital of Jiangxia District, Wuhan, 430200, Hubei, China

^3^ Department of Thoracic Surgery, Zhongnan Hospital of Wuhan University, Wuhan 430071, Hubei, China

* These authors contributed equally to this work.

^#^ Corresponding author: Ming Xu

Department of Thoracic Surgery,

Zhongnan Hospital of Wuhan University,

No.169 Donghu Road, Wuchang District, Wuhan, 430071, China

E-mail: [xumingznhospital@163.com](mailto:xumingznhospital@163.com)


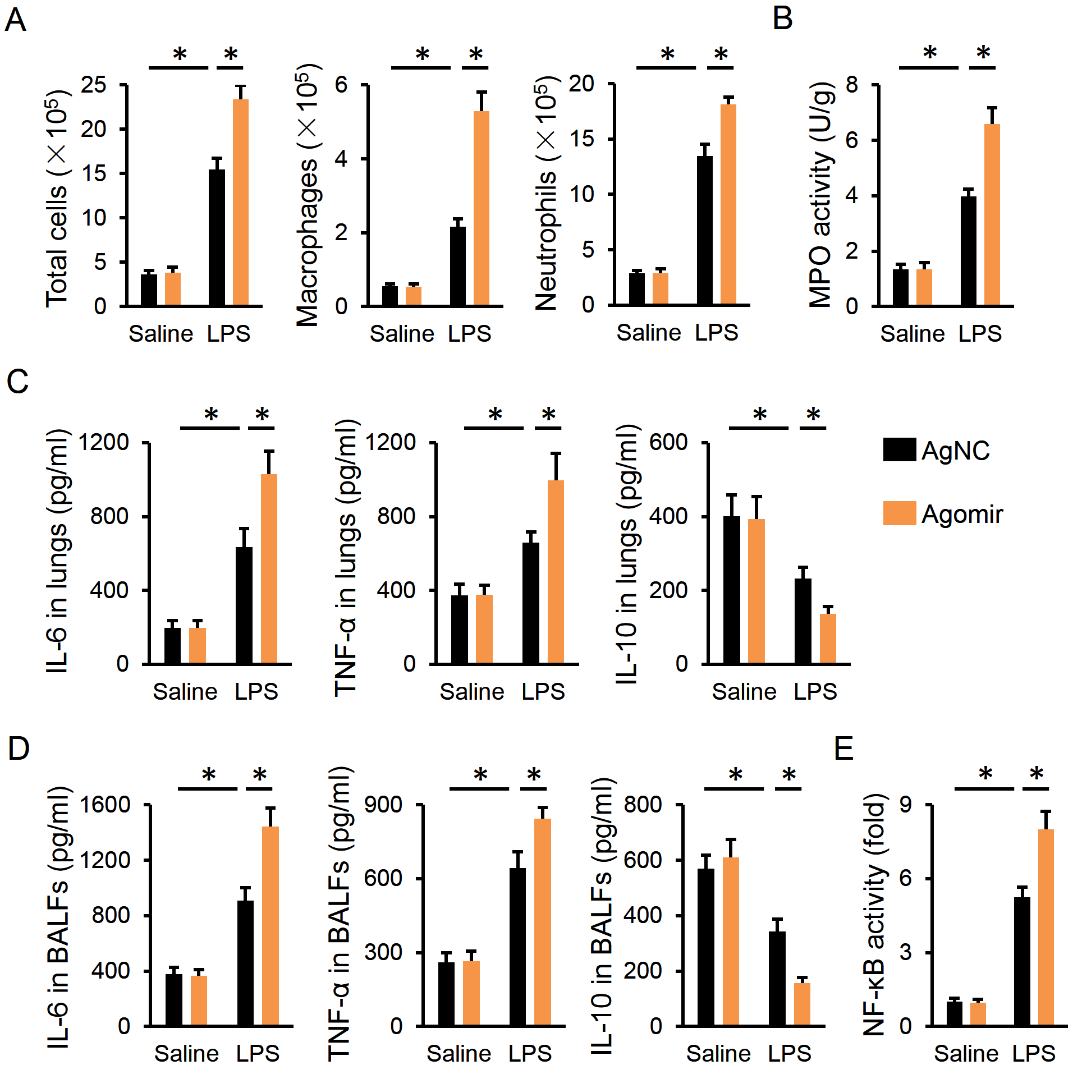


**Fig. S1. MiR-23a-5p agomir aggravates** the **pulmonary inflammation in ALI mice. (A)** Mice were treated with the miR-23a-5p agomir (30 mg/kg/day) or AgNC for 3 consecutive days and then intratracheally injected with 5 mg/kg LPS. 12 h after LPS injection, total cells, macrophages and neutrophils in BALFs were determined. **(B)** MPO activity in the lungs. **(C, D)** The levels of inflammatory makers in the lungs or BALFs. **(E)** Relative NF-κB activity in the lungs. The data are expressed as the means ± SD (n = 6 per group). **P* < 0.05 when compared with the matched group.


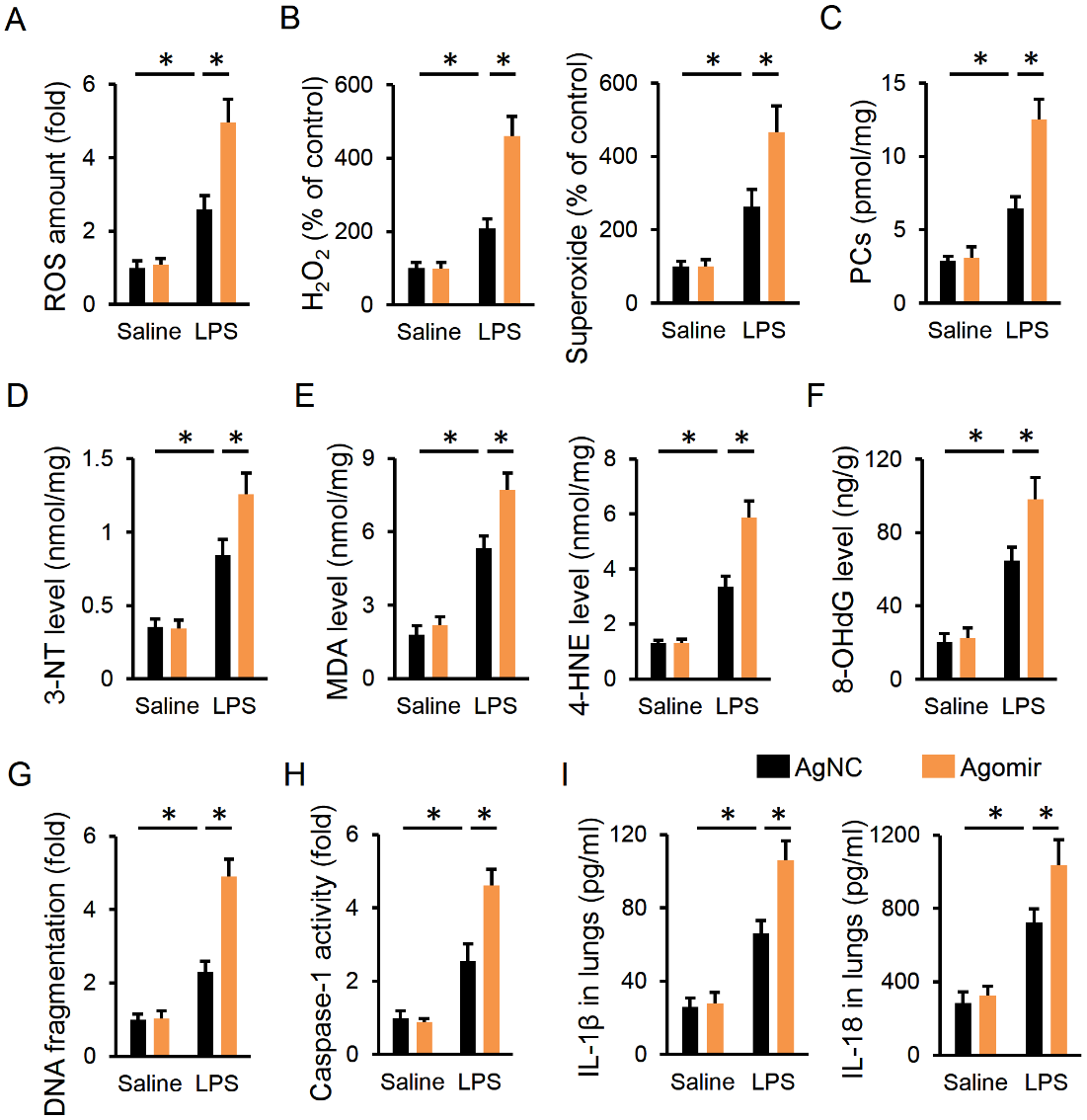


**Fig. S2. MiR-23a-5p agomir increases pulmonary oxidative damage and NLRP3 inflammasome activation in ALI mice. (A)** Intracellular ROS amount. **(B)** Relative levels of H_2_O_2_ and superoxide in the lungs. **(C, D)** Oxidative products from proteins in the lungs. **(E)** Oxidative products from lipids in the lungs. **(F)** Oxidative products from DNA in the lungs. **(G)** Relative level of DNA fragmentation in the lungs. **(H)** Relative caspase-1 activity in the lungs. **(I)** The levels of IL-1β and IL-18 in the lungs. The data are expressed as the means ± SD (n = 6 per group). **P* < 0.05 when compared with the matched group.


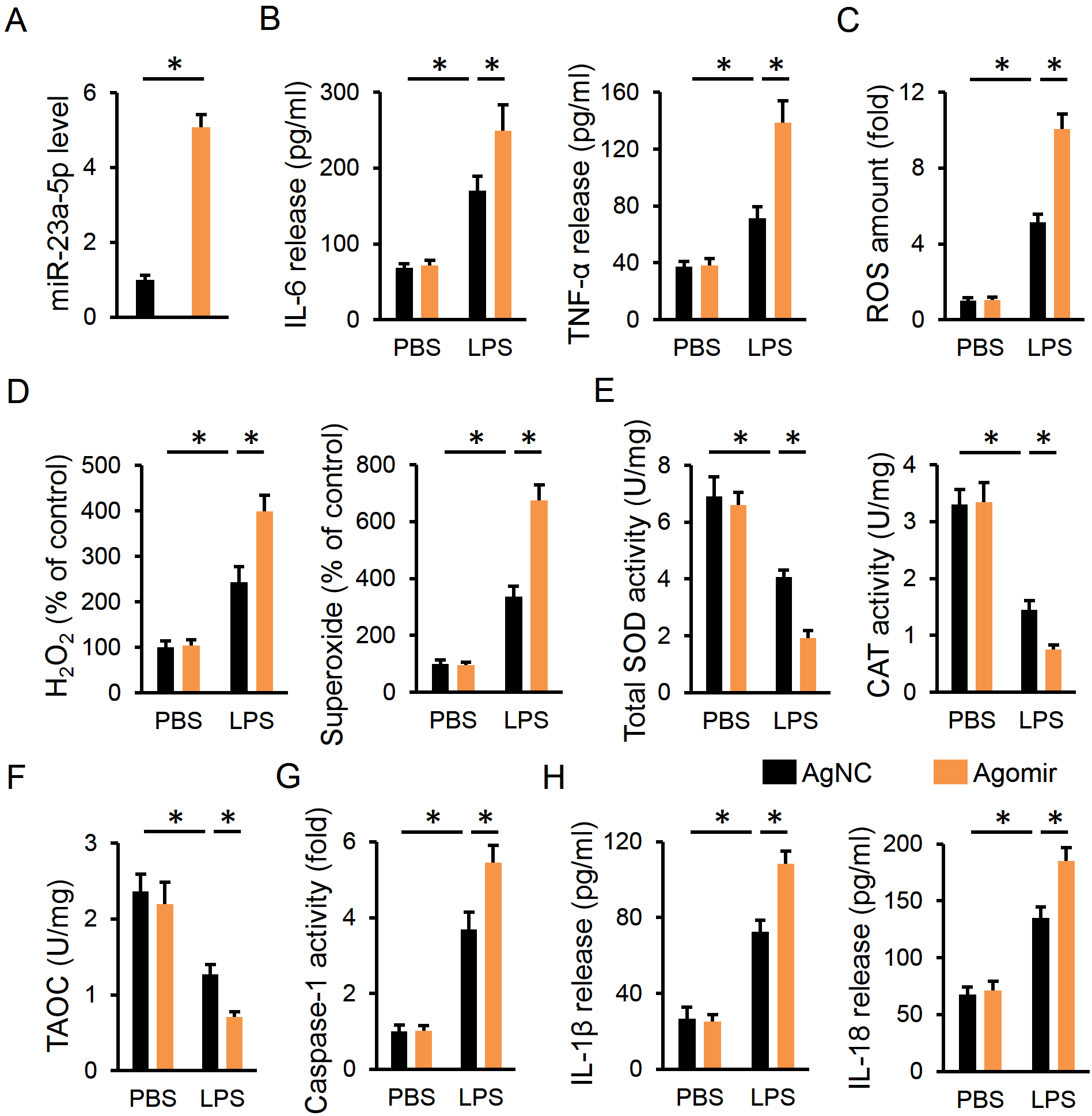


**Fig. S3. MiR-23a-5p agomir promotes LPS-induced inflammation and oxidative stress in macrophages. (A)** Primary macrophages were incubated with the miR-23a-5p agomir (50 nmol/L) or AgNC for 24 h and then incubated in fresh medium for additional 24 h. Relative miR-23a-5p level was detected in primary macrophages. **(B)** Primary macrophages were incubated with the miR-23a-5p agomir (50 nmol/L) or AgNC for 24 h and then incubated in fresh medium for additional 24 h before LPS (100 ng/mL) stimulation for 6 h. The levels of IL-6 and TNF-α in the culture supernatants from LPS-treated macrophages were determined. **(C)** Intracellular ROS amount. **(D)** Relative levels of H_2_O_2_ and superoxide in macrophages. **(E, F)** Cellular anti-oxidant capacity determined by TAOC, total SOD and CAT activities. **(G)** Relative caspase-1 activity in macrophages. **(H)** The levels of IL-1β and IL-18 in the culture supernatants from LPS-treated macrophages. The data are expressed as the means ± SD (n = 6 per group). **P* < 0.05 when compared with the matched group.
